# Effect of Environmental Factors on *Fusarium* Species and Associated Mycotoxins in Maize Grain Grown in Poland

**DOI:** 10.1371/journal.pone.0133644

**Published:** 2015-07-30

**Authors:** Elżbieta Czembor, Łukasz Stępień, Agnieszka Waśkiewicz

**Affiliations:** 1 Grasses and Legumes Department, Plant Breeding and Acclimatization Institute—NRI, Radzików, Poland; 2 Department of Pathogen Genetics and Plant Resistance, Institute of Plant Genetics, Polish Academy of Sciences, Poznań, Poland; 3 Department of Chemistry, Poznań University of Life Sciences, Poznań, Poland; Woosuk University, REPUBLIC OF KOREA,

## Abstract

Maize is one of the most important crops and Poland is the fifth largest producing country in Europe. Diseases caused by *Fusarium* spp. can affect the yield and grain quality of maize because of contamination with numerous mycotoxins produced by these fungi. The present study was performed to identify the prevailing *Fusarium* species and the environmental factors affecting their frequencies and the contamination of grain with the main mycotoxins deoxynivalenol (DON), zearalenone (ZON) and fumonisin B1 (FB1). Thirty kernel samples were collected in three locations in 2011 and in seven locations in 2012 from three hybrids. On average, 25.24% kernels were colonized by *Fusarium* spp. (424 strains were isolated). *Fusarium verticillioides* and *F*. *temperatum* were the most prevalent species, *F*. *subglutinans*, *F*. *proliferatum* and *F*. *graminearum* were in minor abundance. In total, 272 isolates of *F*. *verticillioides* and 81 isolates of *F*. *temperatum* were identified. *Fusarium temperatum* frequency ranged from 1.70% to 28.57% and differences between locations were significant. Fumonisin B1 was found in all tested samples. DON was found in 66.67% and ZON in 43.33% of samples. Rainfall amount positively affected *F*. *temperatum* and *F*. *subglutinans* frequency in opposite to mean temperatures in July. On the other hand, relationships between frequency of these species and historical data from 1950–2000 for annual temperature range were negative in contrast to the coldest quarter temperatures.

## Introduction

Maize is one of the most important crops worldwide and in Europe Poland is the fifth producing country (520 thousand ha for silage and 534 thousand ha for grain in 2012), with an increasing trend towards more production. Maize grain has similar feed value to wheat grain and is much cheaper.

Red and pink ear rots caused by *Fusarium* spp. are important factors affecting the yield and quality of maize grain, mainly because of its contamination with mycotoxins produced by these fungi. An important part of effective crop protection strategy is monitoring the *Fusarium* species associated with maize as well as with the specific environment. In Poland the large, central lowlands are quite flat and narrow in the West, while expanding to the North and South. Recently, the frequency of some *Fusarium* spp. occurrence has shown a tendency to increase in various European countries, including Poland [1]. This tendency is probably related to warmer climate and conservation tillage techniques combined with maize- and wheat-dominated crop rotation systems increasingly practised in these regions [2]. Residues of maize remaining on the soil surface promote the survival of fungal pathogens as well as that of European corn borer larvae, which may enhance the risk of ear infection with *Fusarium* spp. [3–5].

Depending on specific climatic conditions, the dominant *Fusarium* spp. causing red ear rot are: *F*. *graminearum* Schwabe and *F*. *culmorum* (Wm. G. Sm.) Sacc. (producing deoxynivalenol and other trichothecenes as well as zearalenone), followed by *F*. *verticillioides* (Sacc.) Nirenberg and *F*. *proliferatum* (Matsush.) Nirenberg (producing mainly B analogues of fumonisins [6–10]. Less important species include *F*. *subglutinans* (Wollenw. and Reinking) Nelson, Toussoun and Marasas and *F*. *sporotrichioides* Scherb. [8, 11, 12]. Very recently, *F*. *temperatum*, a new species closely related to the *F*. *subglutinans* Group 1 and producing beauvercin, has been described and identified in maize samples from Belgium, Poland and also in Northern China, where climatic conditions are harsh [13–15].

In Poland, *Fusarium* species occurrence and their mycotoxin production have been studied since 1969 [16]. Until the 1990's the climate of this country has been described as temperate with relatively cold winters and warm summers, which is greatly influenced by oceanic air currents from the west, cold polar air from Scandinavia and Russia, as well as warmer, sub-tropical air from the South. During this time, the most frequent *Fusarium* species in small grain cereals, and in maize were *F*. *graminearum* and *F*. *culmorum*. Recently, the climate has become much warmer, with, frequent, day-to-day and year-to-year variability in the weather patterns noted. As a consequence, the occurrence of other species, such as *F*. *verticillioides* or *F*. *temperatum*, began to increase. However, the mean mycotoxin concentration in cereal grain samples collected in Poland was usually lower in comparison to other European countries [17–20].

The contamination of human food and animal feed with mycotoxins causes acute or chronic health problems in humans or livestock, such as equine leukoencephalomalacia, human esophaegal and liver cancer as well as other diseases [8, 21–29]. Strict maximum levels for *Fusarium* mycotoxins (deoxynivalenol, zearalenone and fumonisins) are in place for foodstuffs (EC 2007) and guidance values have been issued for animal feed in the European Union (EC 2006) (http://eur-lex.europa.eu/LexUriServ/LexUriServ.do?uri=OJ:L:2006:234:0035:0040:EN:PDF).

The use of genetic resistance is one of the most effective practices for disease control, and pedigree selection is an effective method to obtain genetic gain for ear rot resistance [30–33]. Another important part of effective crop protection strategy against ear rot is monitoring the *Fusarium* species associated with maize as well as with the specific environment [34]. Several primary infection pathways of maize kernels by *Fusarium* species were identified, having significant effect on the species spectrum [35]. One of the most important infection pathways is through the silk channels during flowering time. Silks are susceptible during the first 6 days after silk emergence and much less susceptible after this time. Spores, dispersed by wind or insect vectors, start to germinate on silks and temperature and humidity are the crucial factors affecting this process. The silk infection pathway is the most common for *F*. *graminearum* and *F*. *culmorum* [36]. Also *F*. *verticillioides* can infect maize via silks, however, other infection pathways, such as damaged kernels and systemic growth of the pathogen play important roles [3, 32, 37].

Therefore, the main goal of this study was to evaluate the naturally occurring *Fusarium* species and associated mycotoxins in grain samples collected from hybrids commonly grown in different regions of Poland, and to identify the environmental factors affecting the species frequency shifts in the populations of these pathogens.

## Materials and Methods

### Maize grain sampling

**Fig 1:**
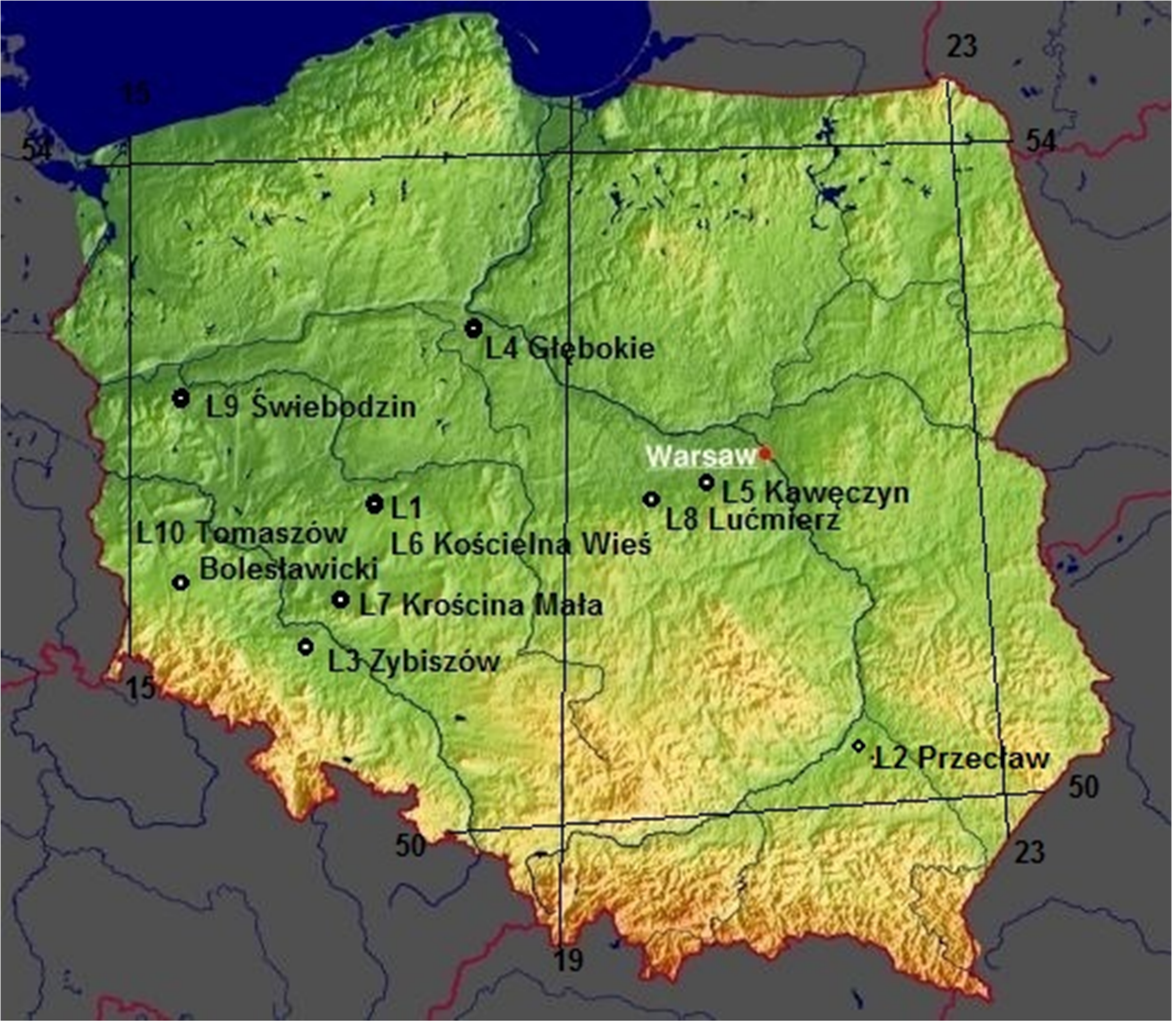
Geographic locations representing different environments in Poland where the field trials were conducted. Geographic locations of the field trials with three maize sampled hybrids (H1-H3) were conducted during 2011 (L1-L3) and 2012 (L4-L10). Samples were collected in regions of Poland with different environmental conditions: Central, Central-Western, South-Eastern and South-Western (GinkoMaps-project; http://www.ginkgomaps.com).

A total of 30 maize grain samples were evaluated for *Fusarium* spp. occurrence. Samples were collected in locations representing regions of Poland with different environmental conditions (Central, Central-Western, South-Eastern and South-Western) across 2011–2012 (Fig 1). In 2011 samples from three locations were evaluated (L1: Kościelna Wieś, L2: Przecław and L3: Zybiszów) and in 2012 samples originated from seven locations (L4: Głębokie, L5: Kawęczyn, L6: Kościelna Wieś, L7: Krościna Mała, L8: Lućmierz, L9: Świebodzin and L10: Tomaszów Bolesławicki) (Table 1, Fig 1). Three maize hybrids, commonly grown in Europe, were sampled in all locations: PR38A79 (H1), Ronaldino (H2) and Ricardino (H3). Additionally, in 2011 in one location (L1) Es Paroli hybrid was sampled instead of PR38A79. Hybrids represented different levels of resistance to ear rot at high disease pressure and under natural infection. Additionally, they represented different maturity groups: Ricardino with FAO 230 (flint type of kernels) belongs to a middle-early group and Ronaldino with FAO 260 (semi-flint type of kernels) and PR38A79 with FAO 270 belong to a middle-late group.

**Table 1:** Historical data (1950–2000) for locations L1-L3 (2011 samples) and L4-L10 (2012 samples) generated using DIVA-GIS.

Variable	Kościelna Wieś (L1, L6)	Przecław (L2)	Zybiszów (L3)	Głębokie (L4)	Kawęczyn (L5)	Krościna Mała (L7)	Lućmierz (L8)	Świebodzin (L9)	Tomaszów Bol. (L10)
**Annual mean temp. [**°**C]**	10.9	10.6	10.8	10.3	10.4	10.8	10.6	11.2	10.6
**Mean monthly temperature range [**°**C]**	8.4	8.7	9.0	7.8	8.1	8.9	8.0	7.5	7.7
Isothermality (2/7) (* 100)	27.6	26.3	30.5	25.7	25.3	29.9	25.1	25.3	27.5
Temperature seasonality (STD * 100)	791.6	857.4	734.2	805.9	855.3	746.7	861.2	781.6	716.3
**Max temp. of warmest month [**°**C]**	26.3	26.8	25.9	25.5	26.1	25.9	26.3	25.9	25.1
**Min temp. of coldest month [**°**C]**	-4.3	-6.2	-3.6	-4.9	-5.7	-3.8	-5.7	-3.6	-3.0
**Temp. annual range (5–6) [**°**C]**	30.6	33.0	29.5	30.4	31.8	29.7	32.0	29.5	28.1
**Mean temp. of wettest quarter [**°**C]**	19.8	19.9	19.1	19.3	19.7	19.2	20.0	18.6	17.4
**Mean temp. of driest quarter [**°**C]**	1.9	0.7	2.7	1.1	0.6	2.5	0.7	2.5	2.5
**Mean temp. of warmest quarter [**°**C]** ^a^	19.8	19.9	19.1	19.3	19.7	19.2	20.0	20.0	18.7
**Mean temp. of coldest quarter [**°**C]** ^b^	0.2	-1.2	1.0	-0.6	-1.3	0.8	-1.3	0.7	1.0
**Annual precipitation [mm]**	523	633	544	584	559	547	567	595	635
**Precipitation of wettest month [mm]**	71	90	77	79	83	77	83	81	79
**Precipitation of driest month [mm]**	19	28	18	21	23	18	24	22	25
**Precipitation sasonality (CV %)**	39.4	41.7	42.4	37.6	42.7	41.0	41.4	32.9	32.6
**Precipitation of wettest quarter [mm]**	200	249	212	220	221	210	224	200	216
**Precipitation of driest quarter [mm]**	68	92	68	81	81	70	82	88	98
**Precipitation of warmest quarter [mm]** *	200	249	212	220	221	210	224	199	215
Precipitation of coldest quarter [mm]**	77	97	75	92	84	77	89	100	112

^a^ third quarter of each year (July, August and September) from tasseling and silking time till physiological maturity and harvesting time of maize hybrids grown in Poland.

^b^ first quarter of each year (January, February and March)

All trials were conducted on experiment stations owned by the Research Centre for Cultivar Testing (COBORU) (http://www.coboru.pl/English/index_eng.aspx) using the best practice appropriate to the respective area. Permission for sampling was granted by COBORU Deputy Director M. Behnke (SI1 and SI2 forms). Plots were sown in three replications for each hybrid to a density of 83 300 plants per hectare. Plot size was 16.32 m^2^ (10.88 m x 1.50 m) with two rows and 75 cm distance between rows, 68 plants per row. Hybrids were randomized within blocks. Experiments were bordered by four rows of maize hybrid. At harvest, kernels from the three replicates were pooled and thoroughly mixed. Representative grain samples of half a kilogram of each hybrid from each locality were collected and stored in a cold room at 4°C and were made available as plant material for the research project HORzg 8421/1 “Plant improvement for sustainable agroecosystems, high-quality food and crop production for non-food purposes” supported by the Polish Ministry of Agriculture and Rural Development. Results of this project are publically available.

### Mycotoxin analyses

Fumonisin B_1_, zearalenone and deoxynivalenol standards were purchased with a standard grade certificate from Sigma-Aldrich (Steinheim, Germany). Sodium dihydrophosphate, potassium chloride, acetic acid and *o*-phosphoric acid were purchased from POCh (Gliwice, Poland). Organic solvents (HPLC grade), disodium tetraborate, 2-mercaptoethanol and all other chemicals were also purchased from Sigma-Aldrich (Steinheim, Germany). Water for the HPLC mobile phase was purified using a Milli-Q system (Millipore, Bedford, MA, USA).

Extraction and purification procedure: 10 g of homogenized ground samples of maize kernels were prepared for analysis. All mycotoxins (FB_1_, ZON, DON) were extracted and purified according to the detailed procedure described earlier [20, 38, 39]. The eluate was evaporated to dryness at 40°C under a stream of nitrogen. Dry residue was stored at -20°C until HPLC analyses.

The chromatographic system utilized a Waters 2695 high-performance liquid chromatograph (Waters, Milford, USA) with the following detectors: (i) Waters 2475 Multi λ Fluorescence Detector (λ_ex_ = 335 nm, λ_em_ = 440 nm) with a X-Bridge column (3.0x100 mm) for FB_1_ analysis; (ii) Waters 2996 Photodiode Array Detector with Nova Pak C-18 column (300x3.9 mm) for DON (λ_max_ = 224 nm) analysis; (iii) Waters 2475 Multi λ Fluorescence Detector (λ_ex_ = 274 nm, λ_em_ = 440 nm) and Waters 2996 Photodiode Array Detector with Nova Pak C-18 column (150x3.9 mm) for ZON analysis. Quantification of mycotoxins was performed by measuring the peak areas at the retention times according to relevant calibration curves. Limits of detection were: 0.001 μg/g for ZON and 0.01 μg/g for DON and FB_1_.

### *Fusarium* species isolation

Fifty six maize kernels were selected randomly from each sample (a total of 168 from each locality). Kernels were soaked in distilled water for 24 hours on a shaker. Afterwards, they were surface-disinfected in ethanol (15 sec) and rinsed 3 times in distilled water, dried on sterile filter paper, placed on water agar (2% of Bacto agar, Difco) in Petri dishes, supplemented with neomycin and streptomycin sulfate (100 mg/L and 200 mg/L, respectively) and incubated at 22°C in darkness. After incubation for 7–12 days each culture was sub-cultured using the single spore technique. Pure cultures of *Fusarium* spp. were grown at 22°C (12 h photoperiod) for 10 days on SNA to produce macroconidia of uniform size and form and on PDA for colony morphology assessment [40].

### Fungal species identification

Genomic DNAs of the fungal strains were extracted and purified using a procedure described previously [41]. The identification of *Fusarium* species among the fungal strains isolated from maize kernels was done using molecular markers and the analysis of diagnostic sequences. In particular, sequence characterized amplified region (SCAR) markers were used for *F*. *proliferatum*, *F*. *subglutinans* and *F*. *verticillioides* species identification [42]. Two primer pairs were used for *F*. *temperatum* identification: one previously described [13] and the second, designed on the basis of the translation elongation factor (*tef*-1alpha) sequence analysis: (Temp1: 5’-CACTCGAGCAATGCGCGTTTCT-3’/Temp2: 5’-CGAATTAAGGGAGAACGAGGCAT-3’). *Fusarium graminearum*, *F*. *equiseti*, *F*. *thapsinum* and *F*. *poae* species were identified based on the sequence analysis of the the translation elongation factor (*tef*-1alpha) gene, amplified and sequenced using the primers and procedures described previously [43]. PCRs were conducted using BioRad C-1000 thermal cyclers in a 20 μl volume. Thermo Scientific Phire II Taq DNA polymerase was used, along with SIGMA dNTPs and custom primers. The PCR conditions were as described previously [43]. Amplified DNA fragments were electrophoresed in 1.5% agarose gels (AppliChem) and 1x TBE buffer (SIGMA).

For sequencing, the amplified fragments of *tef*-1alpha gene were purified using exonuclease I (Thermo Scientific) and Shrimp Alkaline Phosphatase (Thermo Scientific) according to Błaszczyk et al. [44]. Sequence labeling and reading were done using protocols validated previously [43, 45]. Sequence reading was done using Applied Biosystems equipment. Obtained sequences were analysed using Chromas (Technelysium) and MEGA 4 [46] software packages. Species identification was confirmed based on comparison to reference GenBank sequences of the respective species using BLASTn algorithm.

### Meteorological data

**Fig 2:**
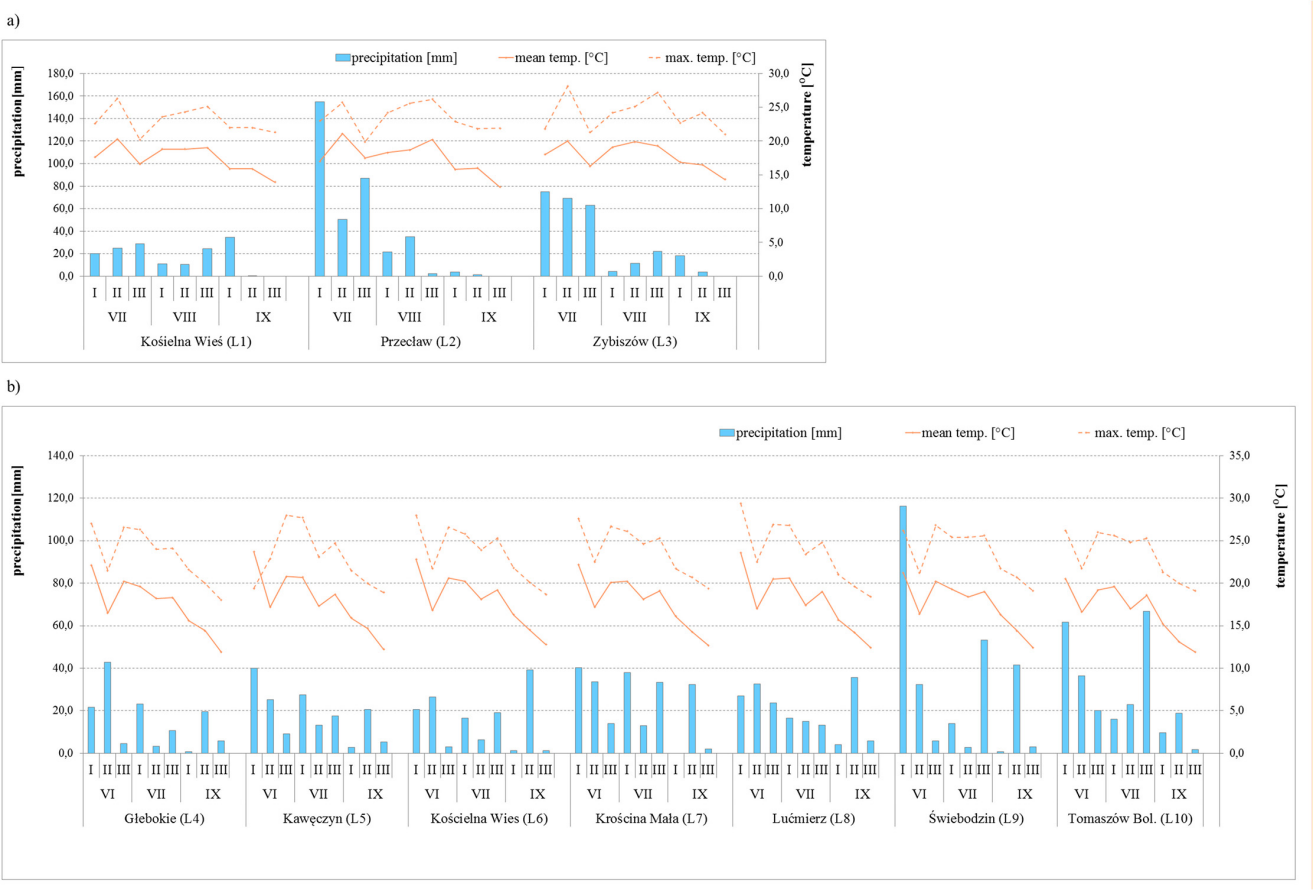
Temperature and precipitation data for locations L1-L3 (a – 2011 samples) and L4-L10 (b – 2012 samples). Mean and maximum decade temperatures and decade precipitation for the third quarter in (a) locations L1-L3 where samples were collected in 2011 as well (b) locations L4-L10 where samples were collected in 2012. Weather conditions such as mean and maximum temperatures [°C] and precipitations [mm] during the third quarter of the 2011 and 2012 years (July, August and September that means from tasseling and silking time till physiological maturity and harvesting time of sampled hybrids) were monitored and analyzed.

Weather conditions were monitored, including mean and maximum temperatures [°C] and precipitation [mm] during the third quarter of 2011 and 2012 (July, August and September). This is from tasseling and silking time until physiological maturity and harvesting of sampled hybrids) (Fig 2). On the other hand, historical representative data for 1950–2000 for each locations were separately computed using the DIVA-GIS software and are presented in Table 1. They include: annual mean temp. [°C], mean monthly temperature range [°C], isothermality (2/7) (* 100), temperature seasonality (STD * 100), max temperature of warmest month [°C], temp. annual range [°C], mean temp. of wettest quarter [°C], mean temp. of driest quarter [°C], mean temp. of warmest quarter [°C], mean temp. of coldest quarter [°C], annual precipitation [mm], precipitation of wettest month [mm], precipitation of driest month [mm], precipitation seasonality (CV %), precipitation of wettest quarter [mm], precipitation of driest quarter [mm], precipitation of warmest quarter [mm], precipitation of coldest quarter [mm]. Under Polish conditions the warmest quarter is usually the third quarter of each year and the coldest quarter is the first quarter of each ear.

### Statistical analysis

Trials with three sampled maize hybrids were conducted using a randomized complete block design with three replicates. The average occurrences of each *Fusarium* species in maize grain samples collected from 3 hybrids in 3 locations in 2011 and 3 hybrids in 7 locations in 2012 were compared individually using fixed analysis of variance. Comparison between hybrids and locations was done using the Fisher least significant difference test. Pearson correlations between *Fusarium* species and between *Fusarium* species and environment (mean temperatures, total rainfall amount latitude, longitude and altitude) were calculated. All statistical analyses were made using the InfoStat software.

## Results

Maize grain samples evaluated for *Fusarium* spp. occurrence in 2011 and 2012 were collected from three hybrids in locations representing regions of Poland with different environmental conditions (Fig 1). Thirty samples were evaluated: 9 samples were collected from hybrids grown in three locations (L1-L3) in 2011 and 21 samples were collected in seven locations (L4-L10) in 2012.

### Environmental conditions

Geographical localization of the sample collection fields were as follows: latitude ranging from 50.1928°N to 52.9818°N, longitude from 15.0776°E to 21.445°E and the altitude from 77 to 196 m above sea level (Fig 1).

Weather data were analyzed separately. These were monitored when the experiments were conducted and kernel samples were collected (third quarter of 2011 and 2012) and historical data generated using DIVA-GIS software (representative for 1950–2000). More differences were observed between locations for temperature, based on the data from the long time period than for 2011 and 2012 (Table 1, Fig 2).

Depending on location, July mean temperatures in 2011 were on average 2°C lower than in 2012. On the other hand, in September mean temperatures were higher in 2011 than in 2012. The highest differences of mean and maximum temperatures were observed in the second half of July (ab. 5°C). In 2011 total rainfall amount ranged from 73.9 to 29.8 mm in July, from 37.7 to 58.6 mm in August and from 5.2 to 34.7 mm in September. Consequently, in 2012 rainfall amounts were from 52.0 to 154.4 mm for July, 37.0 to 105.6 mm for August and from 26.2 to 45.6 mm for September.

Based on historical meteorological data it was possible to observe differences for annual range of temperatures (ab. 5°C), temperature seasonality, temperature annual range (ab. 5°C) and mean temperature of the wettest quarter. Annual precipitation ranged from 523 to 635 mm. Precipitation in the warmest quarter overlapped with precipitation of the wettest quarter, and the differences between locations were of ab. 20 mm with the exception of Przecław (L2), where annual temperature range was 33.0°C and precipitation for the warmest and wettest quarter was 249 mm). In location L10 (Tomaszów Bolesławicki) annual range temperature and temperature seasonality was the lowest and precipitation of the driest quarter higher than in the other one.

### Mycotoxin concentrations

All maize kernel samples contained FB_1_ (Table 2). DON was found in 66.67% and ZON in 43.33% of samples. Fumonisin B_1_ contamination ranged from 59.68 to 1190.33 μg/kg. The average DON level for positive samples was 50.77 μg/kg with a maximum concentration of 90.54 μg/kg. The same samples were also contaminated with ZON—average level for positive samples was 18.39 μg/kg with a maximum concentration of 59.87 μg/kg. Distribution of positive samples indicated that most samples (53.33%) were contaminated with FB_1_ within the range of 300 to 500 μg/kg.

**Table 2:** Frequencies of grain sample contamination with fumonisin B_1_, deoxynivalenol and zearalenone from 2011 and 2012.

Toxin	Positive samples (%)	Range of contamination (μg/kg)	Average level for positive samples (μg/kg)
**FB**_**1**_	100.00	59.68–1190.33	373.50
**DON**	66.67	n.d. – 90.54	50.77
**ZON**	43.33	n.d. – 59.87	18.39

n.d.—not detected

**Fig 3:**
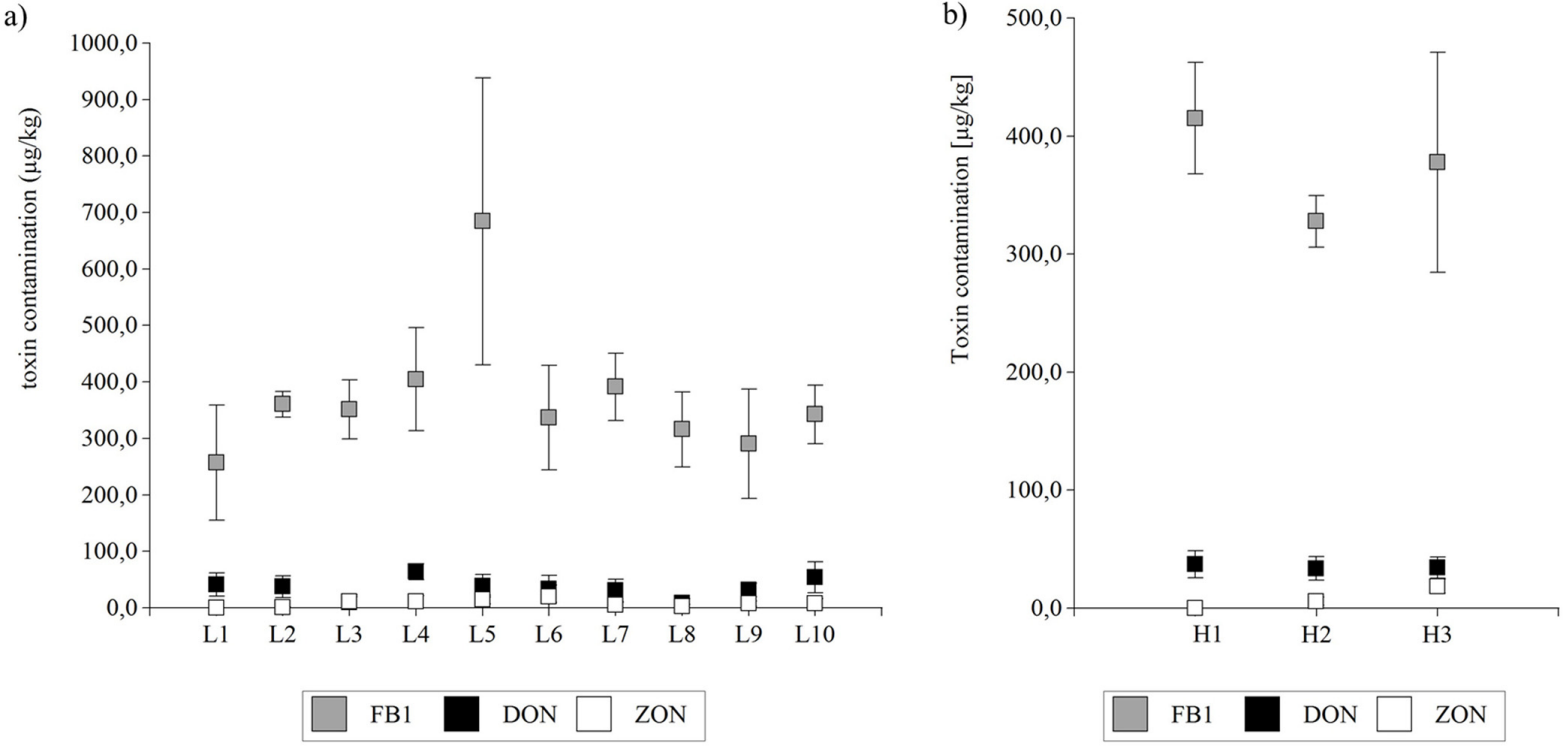
Fumonisin B_1_, deoxynivalenol and zearalenone contamination for grain samples from 2011 and 2012. Fumonisin B_1_ (FB_1_), deoxynivalenol (DON) and zearalenone (ZON) contamination for grain samples representing (a) the average for locations L1-L10 sampled in 2011 and 2012 as well (b) the average for three hybrids H1-H3 sampled in 2011 and 2012 were measured. Bars represent standard error on the probability level 0.05; non-overlapping notches show a significant difference. There were no significant differences in concentrations between years or locations except for ZON. Differences were found between hybrids for ZON content (F = 5.71; p value = 0.0086) (Fig 3B).

On average, the differences in sample contamination with FB_1_ ranged between locations from 257.03 (L1, Kościelna Wieś, Central Poland) to 684.63 μg/kg (L5, Głębokie, Central Poland) (Fig 3A). The highest average level of DON was found in the grain samples from Głębokie (L4) (64.16 μg/kg) and the lowest—from (Zybiszów (L3) (below the limit of detection—LOD). In the case of ZON, the differences between locations were from ≤LOD (Zybiszów, L3) to 19.96 μg/kg (Kościelna Wieś, L6). The average DON levels varied between the hybrids ranging from 33.69 μg/kg (H2, middle-late genotype) to 37.20 μg/kg (H1, middle-late genotype), while the differences for ZON content ranged from ≤LOD (H1, middle-late genotype) to 18.50 μg/kg (H3, middle-early genotype), and the differences for FB_1_ content ranged from 327.76 μg/kg (H2, middle-late genotype) to 414.99 μg/kg (H1, middle-late genotype) (Fig 3B).

### *Fusarium* spp. frequencies

**Fig 4:**
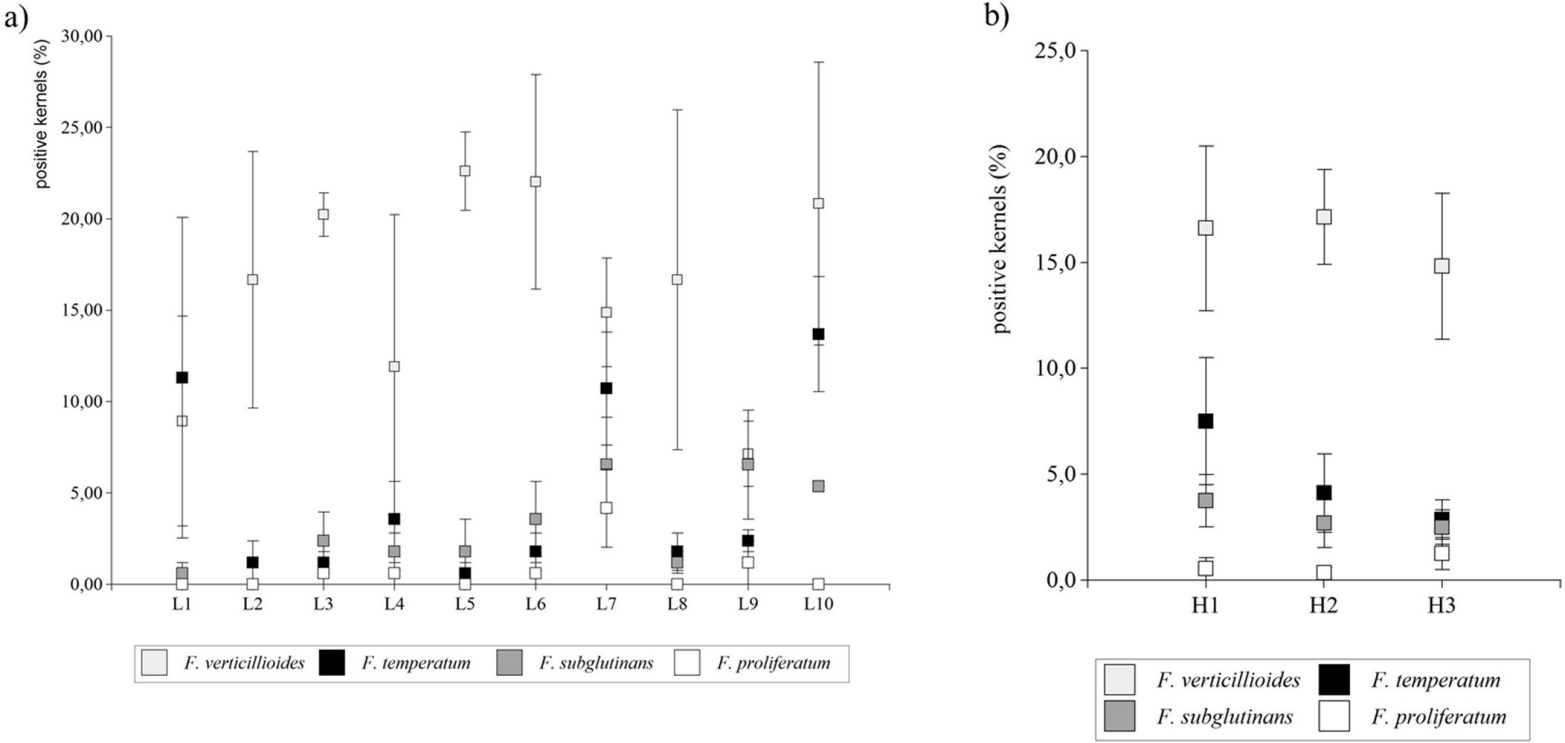
Percentages of kernels containing *Fusarium* spp. in grain samples collected in 2011 and 2012. The most frequent *Fusarium* spp. in grain samples representing (a) the average for locations L1-L3 sampled in 2011 as well (b) the average for three hybrids H1-H3 sampled in 2011 and 2012. Bars represent standard error on the probability level 0.05; non-overlapping notches show a significant difference.

On average, 25.24% of kernels from grain samples collected in three locations in 2011 (L1-L3) and in seven locations in 2012 (L4-L10) were colonized by *Fusarium* spp. (424 strains were isolated from 11680 kernels evaluated) (Table 3). Differences between locations for the *Fusarium* spp. presence ranged from 17.30% (Świebodzin, L9, Central-Western part of Poland) to 40.50% (Tomaszów Bolesławicki, L10, South-Western part of Poland). Eight *Fusarium* species were isolated and identified. Generally, *Fusarium verticillioides* and *F*. *temperatum* were found in all locations. *Fusarium subglutinans* was present in samples collected from nine locations (Table 3, Fig 4).

**Table 3:** Percentages of kernels containing *Fusarium* species collected from H1-H3 hybrids in 2011 and 2012.

Year	Location	Hybrid	*F*. *verticillioides*	*F*. *temperatum*	*F*. *subglutinans*	*F*. *proliferatum*	*F*. *equiseti*	*F*. *graminearum*	*F*. *poae*	*F*. *thapsinum*	Total
**2011**	**L1 (Kościelna Wieś)**	**H1**	-^a^	28.57 (16)	1.79 (1)	-	-	-	-	-	30.36 (17)
	**L1 (Kościelna Wieś)**	**H2**	19.64 (11) ^b^	-	-	-	-	-	-	-	19.64 (11)
	**L1 (Kościelna Wieś)**	**H3**	7.14 (4)	5.36 (3)	-	-	-	-	-	-	12.50 (7)
	**L2 (Przecław)**	**H1**	30.36 (17)	3.57 (2)	-	-	-	-	-	-	33.93 (19)
	**L2 (Przecław)**	**H2**	12.50 (7)	-	-	-	-	-	-	-	12.50 (7)
	**L2 (Przecław)**	**H3**	7.14 (4)	-	-	-	-	-	-	-	7.14 (4)
	**L3 (Zybiszów)**	**H1**	21.43 (12)	1.79 (1)	5.36 (3)	-	-	-	-	-	28.57 (16)
	**L3 (Zybiszów)**	**H2**	17.86 (10)	-	-	1.79 (1)	-	-	-	-	19.64 (11)
	**L3 (Zybiszów)**	**H3**	21.43 (12)	1.79 (1)	1.79 (1)	-	-	-	-	-	25.00 (14)
**2012**	**L4 (Głębokie)**	**H1**	3.57 (2)	-	1.79 (1)	-	-	-	-	-	5.36 (3)
	**L4 (Głębokie)**	**H2**	3.57 (2)	7.14 (4)	-	-	-	-	-	1.79 (1)	12.50 (7)
	**L4 (Głębokie)**	**H3**	28.57 (16)	3.57 (2)	3.57 (2)	1.79 (1)	-	-	-	-	37.50 (21)
	**L5 (Kawęczyn)**	**H1**	19.64 (11)	-	-	-	-	-	-	-	19.64 (11)
	**L5 (Kawęczyn)**	**H2**	26.79 (15)	1.79 (1)	5.36 (3)	-	-	-	-	1.79 (1)	35.71 (20)
	**L5 (Kawęczyn)**	**H3**	21.43 (12)	-	-	-	-	-	-	-	21.43 (12)
	**L6 (Kościelna Wieś)**	**H1**	30.36 (17)	3.57 (2)	7.14 (4)	-	-	-	5.36 (3)	-	46.43 (26)
	**L6 (Kościelna Wieś)**	**H2**	25.00 (14)	1.79 (1)	-	1.79 (1)	-	-	-	-	28.57 (16)
	**L6 (Kościelna Wieś)**	**H3**	10.71 (6)	-	3.57 (2)	-	-	-	-	-	14.29 (8)
	**L7 (Krościna Mała)**	**H1**	17.86 (10)	10.71 (6)	1.79 (1)	5.36 (3)	-	-	-	-	35.71 (20)
	**L7 (Krościna Mała)**	**H2**	17.86 (10)	16.07 (9)	10.71 (6)	-	-	-	-	-	44.64 (25)
	**L7 (Krościna Mała)**	**H3**	8.93 (5)	5.36 (3)	7.14 (4)	7.14 (4)	-	-	-	-	28.57 (16)
	**L8 (Lućmierz)**	**H1**	32.14 (18)	3.57 (2)	1.79 (1)	-	-	-	-	-	37.50 (21)
	**L8 (Lućmierz)**	**H2**	17.86 (10)	-	1.79 (1)	-	1.79 (1)	3.57 (2)	-	-	25.00 (14)
	**L8 (Lućmierz)**	**H3**	-	1.79 (1)	-	-	-	-	-	-	1.79 (1)
**2012**	**L9 (Świebodzin)**	**H1**	3.57 (2)	3.57 (2)	12.50 (7)	-	-	-	-	-	19.64 (11)
	**L9 (Świebodzin)**	**H2**	8.93 (5)	1.79 (1)	3.57 (2)	-	-	-	-	-	14.29 (8)
	**L9 (Świebodzin)**	**H3**	8.93 (5)	1.79 (1)	3.5 (2)	3.57(2)	-	-	-	-	17.86 (10)
	**L10 (Tomaszów Bol.)**	**H1**	7.14 (4)	19.64 (11)	5.3 (3)	-	-	-	-	-	32.14 (18)
	**L10 (Tomaszów Bol.)**	**H2**	21.43 (12)	12.50 (7)	5.36 (3)	-	-	-	-	-	39.29 (22)
	**L10 (Tomaszów Bol.)**	**H3**	33.93 (19)	8.93 (5)	5.36 (3)	-	-	-	1.79 (1)	-	50.00 (28)
**Total (L1—L10)** ^c^			16.19 (272)	4.82 (81)	2.98 (50)	0.71 (12)	0.06 (1)	0.12 (2)	0.24 (4)	0.12 (2)	25.24 (424)
**LSD Fisher, 0.05** ^d^			ns	8.823	ns	2.26	ns	ns	ns	ns	ns
**Positive samples (%)**			93.33	70.00	63.33	20.00	3.33	3.33	6.67	6.67	100

^a^ not detected.

^b^ the numbers of positive kernels are in parentheses.

^c^ average for 30 grain samples (collected from 3 hybrids in 10 environments)

^d^ after rejection of the hypothesis of the lack of differences between environments using analysis of variance at a significance level of 0.05, their comparison was done using Fisher least significant difference test; differences between hybrids were not significant.

*Fusarium verticillioides* was the prevailing species detected in 16.19% of kernels (272 strains were isolated) and its frequence varied from 7.14% (Świebodzin, L9; Central-Western part of the country) to 22.62% (Kawęczyn, L5; Central Poland). The incidence of *F*. *verticillioides* isolates in grain samples originating from H1 (middle-late genotype) ranged from 0% to 30.36%, in grain samples originating from H2 from 3.57% to 25.00% and in middle-early genotype (H3) grain samples from 7.14% to 33.93%. *Fusarium temperatum* frequency ranged from 0.60% (Kawęczyn, L5) to 13.69% (Tomaszów Bolesławicki, L10). Percentages of kernels containing *F*. *temperatum* in grain sampled from the hybrids ranged from 2.86% (H1) to 7.50% (H2). Depending on the environment, the percentage of kernels sampled from H1 (belonging to middle-late group) ranged from 0% to 28.57% (7.50% on average), in grain sampled from H2 (middle-late group) from 0% to 16.07%. (4.11% on average). In grain sampled from H3 (representing middle-early group) the incidence of *F*. *temperatum* was lower than in the samples collected from hybrids belonging to the middle-late group and it ranged from 0% to 8.93%.

*Fusarium subglutinans* colonized 2.98% of tested kernels (50 strains were isolated). When locations were compared, *F*. *subglutinans* frequencies ranged from 0% (Przecław, L2, South-Eastern Poland) to 6.55% (Głębokie, L4, Central Poland). On average, 3.75% of kernels sampled from H1, 2.68% of kernels sampled from H2 and 2.50% of kernels sampled from H3 contained isolates of this species.

*Fusarium graminearum* isolates were present in grain samples from one environment only (Lućmierz, L8, Central Poland) and exclusively in grain collected from one hybrid (H2). Similarly, *F*. *equiseti* was found only in (Lućmierz, L8, H2 hybrid) and *F*. *poae* only in (Kościelna Wieś, L6, H1 hybrid). Two *F*. *thapsinum* isolates were found in samples from Głębokie (L4) and Kawęczyn (L5). There were no differences in species occurrence between years, locations or hybrids, with the exception of *F*. *temperatum* (F = 2.73, p value = 0.05) and *F*. *proliferatum* (F = 2.31, p value = 0.05) (Fig 4).

### Effect of environmental conditions on toxins content and *Fusarium* spp. frequency

Relationships between the most frequent *Fusarium* species (*F*. *verticillioides*, *F*. *temperatum* and *F*. *subglutinans*), geographic location where experiments were conducted (latitude, longitude and altitude) and meteorological conditions in a short time period (2011–2012) and long time period (historical data representative for 1950–2000) were calculated using Pearson correlations.

**Fig 5:**
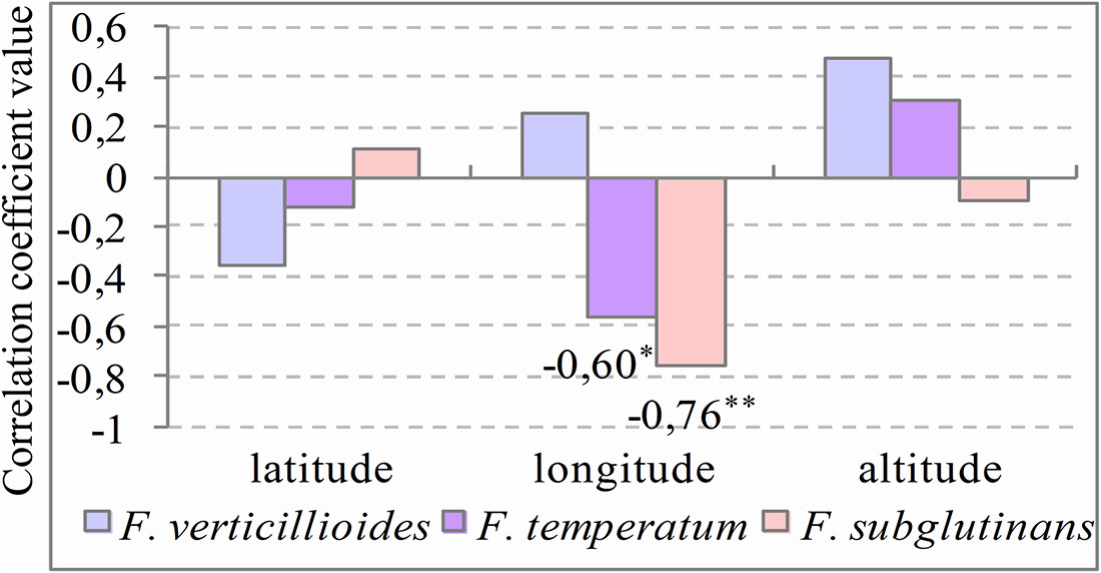
Correlation coefficients between the presence of *F*. *verticillioides*, *F*. *temperatum* and *F*. *subglutinans*, and geographic location. Correlation coefficients between percentages of kernels containing *F*. *verticillioides*, *F*. *temperatum* and *F*. *subglutinans* in grain sampled from 3 hybrids in 2011 and 2012 and geographic localization of the locations where experiments were conducted (latitude, longitude and altitude; *—significance level of 0.05; **—significance level of 0.01).

The correlation analysis confirmed that in the eastern part of the country *F*. *temperatum* and *F*. *subglutinans* frequencies were lower than in the western part–correlation coefficient between longitude and *F*. *subglutinans* frequency was negative (r = -0.76, p = 0.01) similarly as for *F*. *temperatum* frequency (r = -0.60, p = 0.05) (Fig 5).

Additionally, it confirmed that only mean and maximum temperatures of the second half of July and mean temperature of the second half of September negatively correlated with *F*. *subglutinans* frequency (r = -0.60, p = 0.05, to r = 0.66, p = 0.01 and r = -0.67, p = 0.01, respectively). Correlation coefficient between rainfall amount in third decade of August and the percentage of kernels containing *F*. *temperatum* and *F*. *subglutinans* was positive (r = 66, p = 0.01 and r = 0.80, p = 0.01, respectively) as well as for the rainfall amount during second half of September and *F*. *subglutinans* frequency (r = 0.65, p = 0.01).

**Fig 6:**
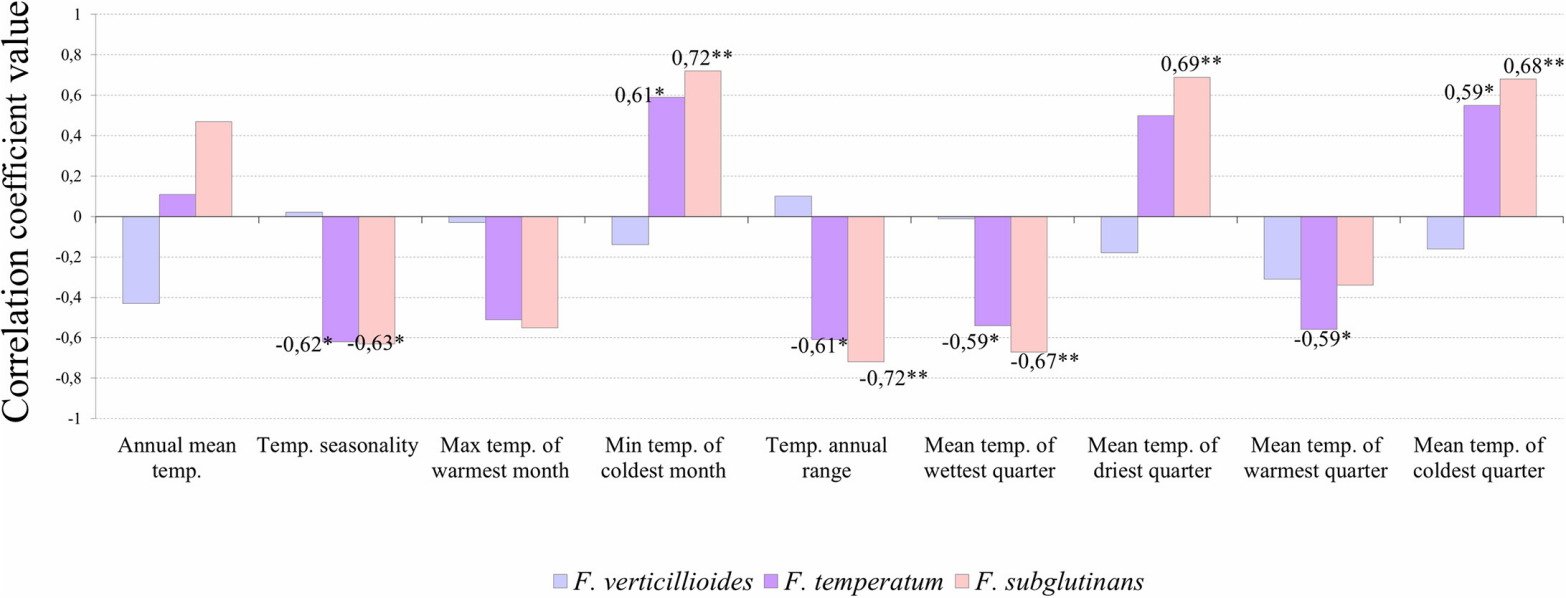
Correlation coefficients between the presence of *F*. *verticillioides*, *F*. *temperatum* and *F*. *subglutinans* and historical temperature data. Correlation coefficients between percentages of kernels containing *F*. *verticillioides*, *F*. *temperatum* and *F*. *subglutinans* in grain samples collected in 2011 and 2012 and historical temperature data (representative for 1950–2000; generated using the DIVA-GIS software; *—significance level of 0.05; **—significance level of 0.01).

Higher correlations were found between historical data representing temperatures in a long time period and *F*. *temperatum*, as well as *F*. *subglutinans* frequencies (Fig 6). Temperature seasonality negative correlated with both frequencies (r = -0.62, p = 0.05 and r = -0.63, p = 0.05, respectively) as well as the annual range of temperatures (r = -0.61, p = 0.05 and r = -0.72, p = 0.01, respectively) or mean temperatures of wettest quarter (r = -0.59, p = 0.05 and r = -0.67, p = 0.01, respectively). Mean temperatures of coldest quarter positively correlated with both *F*. *temperatum* and *F*. *subglutinans* incidence.

## Discussion

Temperature and rainfall are the main factors affecting the development of *Fusarium* species causing important diseases of maize and other small grain cereals. However, the impact of these climatic factors is influenced by other environmental variables, such as additional fungal diseases, drought stress or host-dependent factors [47, 48]. Therefore, the creation of an effective crop protection strategy should involve many steps, such as monitoring the environment and the previous crop during growing periods on both the macro and the micro scale [34].

Poland is a country where the large, central lowlands are quite flat and narrow in the West, while expanding to the North and South. The region is cut by two major rivers, including the Oder which defines the area of West-Central Poland and the Vistula, which defines the lowland areas of East-Central Poland. Using historical meteorological data it was possible to observe differences between particular geographical locations regarding such variables as annual temperature range (ab. 5°C), temperature seasonality, temperature annual range (ab. 5°C) or mean temperature of the wettest quarter and annual precipitation or precipitation of the wettest quarter. For a short time period, the differences for temperatures were not as great as the differences in rainfall amount. This variation plays an important role as a factor influencing the frequencies of species causing ear rot in maize: *F*. *verticillioides*, *F*. *proliferatum*, *F*. *subglutinans* and *F*. *temperatum* (Table 2). The incidence of *F*. *graminearum* recorded for all samples tested was very low, however, the occurrence of DON and ZON in the grain could suggest that the species (or perhaps *F*. *culmorum*) should be present in the kernels. There are many reports stating that *F*. *graminearum* frequency varies significantly among years and locations in many geographical areas [8, 10, 28, 47], however, it is regarded as a predominant species in Central and Northern Europe, *e*.*g*. in Poland [17], Austria [49, 50], Czech Republic [51] or Slovenia [52], while *F*. *verticillioides* prevails in Southern European countries, such as Italy or Spain [53–57]. Recently climate variability determines the *Fusarium* species population variability and the distribution of *F*. *verticillioides* in countries of Central Europe [58–61]. Marin et al. [62] and Torres et al. [63] have reported that optimal temperature for *F*. *verticillioides* germination and growth is about 25°C. Reid et al. [36]. found that *F*. *verticillioides* germinates and grows over a broader range of temperatures and water activities than *F*. *graminearum*. The minimum temperature required for *F*. *verticillioides* growth is 4°C and for *F*. *graminearum* it is 10°C [64]. Therefore, in the presence of the two species, *F*. *verticillioides* has the opportunity to eventually out-compete *F*. *graminearum*. The method of fungal species isolation used in the present study does not favor any of the species present in the samples, nevertheless it allows to detect only the pathogen that is still viable. Yet, it was not possible to detect any *F*. *graminearum* that was already dead. This could explain the presence of the mycotoxins typical for *F*. *graminearum* (DON and ZON) in the grain, while the species was usually not identified as viable pathogen in the harvested grain. In fact, the pathogen may be still present and detectable in the environment, while it is not able to survive in harvested and dried maize cobs. This hypothesis, however, needs to be verified using different experimental approach, utilizing for example monitoring of *F*. *graminearum* presence in the air or maize plant debris.

Another factor, possibly contributing to the low incidence of *F*. *graminearum*, is low water content in plant material at harvest. The minimum water activity needed for growth of *F*. *verticillioides* is 0.86 a_w_ and for *F*. *graminearum* it is 0.935. At 25°C, *F*. *verticillioides* spores are able to germinate at 0.88 a_w_ [62–63], and *F*. *graminearum* spores germinate at 0.94 a_w_ [65]. It might explain the lower incidence of *F*. *verticillioides* on the flat area of central and West-Central part of Poland with latitude higher than 52°N, whereas it was higher in the South of Poland with higher altitudes and with latitude lower than 51.5°N.

The amount of fumonisin B_1_ in maize grain corresponded to the occurrence of *F*. *verticillioides*, however, the mycotoxin content was much lower than reported for the Southern Europe, *e*.*g*. for Spain, [54, 66] or Italy [57, 67]. The levels of maize grain sample contamination with ZON and DON were low. Because of the low *F*. *graminearum* frequency in the grain, one of the possible explanations is that the fungus was present in the ears rachis, however, this part of the cob was not available to be analyzed. Optimal temperatures for fumonisin biosynthesis are between 25 and 30°C [68–70] and DON is usually produced more rapidly at 25°C [71]. Under Polish conditions, mean and maximum temperatures during yield formation and grain ripening stages are usually lower than 25°C and this can be the additional factor influencing lower DON content in maize grain samples.

The second most abundant species was *F*. *temperatum*, a new pathogen described recently [13] and closely related to *F*. *subglutinans*. It occurred in 70% of tested samples (4.82% of all kernels tested), which was higher than for *F*. *subglutinans*. Unlike *F*. *verticillioides*, the frequencies of both species were higher in the environments with mean temperatures of 18°C or lower. Both of them seem to be adapted to similar climatic conditions and a positive correlation between their frequencies was found. Moreover, this confirms the results obtained in Belgium by Scauflaire et al. [13, 29].

The present study corresponds to previous ones, demonstrating low level of ear rot severity on hybrids commonly grown under Polish conditions, based on the phenotypic evaluation of disease symptoms under natural infection in three environments [33]. The level of mycotoxin content in the samples from hybrids tested was low, however, in some of them the contamination with fumonisins and DON was higher than the EU limits. It was found that genotype maturity has a significant effect on DON and FBs concentration in grain. When mean daily temperatures during the growing season exceed 20°C, the early grain varieties take between 80 and 110 days to mature and the medium varieties—110 to 140 days. When mean daily temperatures are below 20°C, there is an extension in 'days to maturity' period, and at 15°C, maize grain needs 200 to 300 days to mature. Furthermore, the level of toxins was lower in grain sampled from hybrids belonging to the early and middle-early groups, which are mostly flint or semi-flint groups, than in grain sampled from hybrids belonging to the dent or semi-dent forms, which are late, with FAO number higher than 250. The frequency of *F*. *verticillioides* in grain sampled from hybrids with semi-flint type of kernels belonging to the middle-late group was higher than for kernels sampled from the middle-early genotype with flint type of kernels, especially from the southern part of Poland, in locations with higher temperatures during tasseling and silking. A similar tendency was observed for the frequency of *F*. *temperatum*, which was more common in locations with lower temperatures, where the climate is more similar to that found in Germany. This finding confirms the results obtained by Robertson et al. [72], however, it does not match their conclusion stating that flint genotypes are generally more susceptible to *F*. *graminearum* and *F*. *verticillioides* infection than dent genotypes in the early maturity group. It is probably caused by the fact that such forms were bred for Northern Europe and the natural selection pressure towards resistant genotypes was low [47, 48, 73]. The present study demonstrated the diversity of *Fusarium* populations in maize grains in relation to different environmental conditions including geographical localization, meteorological data in a short time period and in a long time period. It was possible to observe, how important a role the historical data play for temperature and precipitation level during the short time period. However, additional studies are needed to explain the co-occurrence of DON, ZON and FBs in grain in relation to species populations present, and to create the optimum risk assessment system for forecasting the ear rot and mycotoxin content in maize genotypes specific for Poland and, more generally, for Central Europe.

## Supporting Information

S1 File Permission for sampling in 2011. (PDF)

S2 File Permission for sampling in 2012. (PDF)
