# Supplementary material for: Effect of Environmental Factors on Fusarium Species and Associated Mycotoxins in Maize Grain Grown in Poland
Source: PLoS One. 2015 Jul 30;10(7):e0133644. doi: 10.1371/journal.pone.0133644 (PMC4520617; doi:10.1371/journal.pone.0133644)
Supplement: S1 File — (PDF) [file pone.0133644.s001.pdf]

CENTRALNY OŚRODEK BADANIA ODMIAN ROŚLIN UPRAWNYCH
RESEARCH CENTRE FOR CULTIVAR TESTING
PL 63-022 SŁUPIA WIELKA, tel.: (+48) 61 285 23 41, faks: (+48) 61 285 35 58, e-mail:sekretariat@coboru.pl
www.coboru.pl

Słupia Wielka,13.09.2011
NRK 601/6 /11

Pani Doktor
Elżbieta Kochańska-Czembor
Instytut Hodowli i Aklimatyzacji Roślin
Państwowy Instytut Badawczy
Radzików
05-870 BŁONIE

W odpowiedzi na Pani pismo z dnia 9.09.2011 w sprawie prób ziarna kukurydzy z doświadczeń porejestrowych do badań na zawartość mikotoksyn, COBORU wyraża zgodę na ich udostępnienie.

W roku 2011 zleciliśmy przygotowanie i wysłanie do IHAR Radzików prób ziarna kukurydzy z trzech doświadczeń (Kościelna Wieś, Przecław, Zybiszów), w których uczestniczy między innymi część odmian wpisanych do Krajowego rejestru oraz odmiany z katalogu UE pozytywnie zweryfikowane w doświadczeniach rozpoznawczych; w sumie 39 odmian z trzech grup wczesności.

Próby ziarna zostaną wysłane po zbiorze na koszt odbiorcy.

W przypadku nieudania się doświadczenia w którejś z w/w miejscowości, próby zostaną pobrane z innego doświadczenia.

Zgodnie z deklaracją Pani Doktor, wyniki oceny zawartości toksyn fuzaryjnych zostaną wykorzystane w opracowaniach COBORU, w celu poszerzenia charakterystyk odmian kukurydzy.

Z poważaniem

Z-ca DYREKTORA
ds. badawczo-doświadczalnych
mgr inż. Marcin Behnke

<u>Do wiadomości</u>:
SDOO Przecław
SDOO Zybiszów
ZDOO Kościelna Wieś
<u>Załącznik</u>:
Wykaz odmian

Załącznik

Wykaz odmian kukurydzy w doświadczeniach porejestrowych 2011

Grupa wczesna

1. DKC2971
2. ES Kongress
3. ES Zizou
4. Laureen
5. Lokata
6. NK Ravello
7. Podium
8. Rywal
9. SY Cooky
10. Nerissa *
11. NK Falkone *

Grupa średniowczesna

1. Ambrosini
2. ES Palazzo
3. ES Paroli
4. NK Nekta
5. Prollix
6. Ricardinio
7. Smolan
8. Smolitop
9. SY Multitop
10. MAS 20.F*
11. P8000*
12. PR39D23 *
13. Tiberio *

Grupa średniopóźna

1. Alduna
2. Amoroso
3. DKC3420
4. ES Chrono
5. Geoxx
6. KWS 5133 ECO
7. Lavena
8. MAS 29H
9. NK Eagle
10. PR38N86
11. Ronaldinio
12. Sumas
13. Crispi*
14. Friedrixx*
15. PR38A79 *

* – odmiana z katalogu UE w doświadczeniach porejestrowych
