# Supplementary material for: Effect of Environmental Factors on Fusarium Species and Associated Mycotoxins in Maize Grain Grown in Poland
Source: PLoS One. 2015 Jul 30;10(7):e0133644. doi: 10.1371/journal.pone.0133644 (PMC4520617; doi:10.1371/journal.pone.0133644)
Supplement: S2 File — (PDF) [file pone.0133644.s002.pdf]

CENTRALNY OŚRODEK BADANIA ODMIAN ROŚLIN UPRAWNYCH
RESEARCH CENTRE FOR CULTIVAR TESTING
PL 63-022 SŁUPIA WIELKA, tel.: (+48) 61 285 23 41, faks: (+48) 61 285 35 58, e-mail:sekretariat@coboru.pl
www.coboru.pl

Słupia Wielka, 20.09.2012
NRK 1/8/2012

Pani Doktor
Elżbieta Kochańska-Czembor
Instytut Hodowli i Aklimatyzacji Roślin
Państwowy Instytut Badawczy
Radzików
05-870 BŁONIE

W odpowiedzi na Pani pismo z dnia 17.09.2012 w sprawie prób ziarna kukurydzy z doświadczeń porejestrowych do badań na zawartość mikotoksyn, COBORU wyraża zgodę na ich udostępnienie.

Zgodnie z naszymi ustaleniami zleciliśmy przygotowanie i wysłanie do IHAR Radzików prób ziarna kukurydzy z jedenastu doświadczeń PDOiR, w których uczestniczy między innymi część odmian wpisanych do Krajowego rejestru oraz odmiany z katalogu UE pozytywnie zweryfikowane w doświadczeniach rozpoznawczych; w sumie 33 odmiany (2 miejscowości) oraz 9 odmian (9 miejscowości) z trzech grup wczesności.

Próby ziarna zostaną wysłane po zbiorze na koszt odbiorcy.

W przypadku nieudania się doświadczenia w którejś z w/w miejscowości, próby zostaną pobrane z innego doświadczenia.

Zgodnie z deklaracją Pani Doktor, wyniki oceny zawartości toksyn fuzaryjnych zostaną wykorzystane w opracowaniach COBORU, w celu poszerzenia charakterystyk odmian kukurydzy.

Z poważaniem

Z-ca DYREKTORA
ds. badawczo-doświadczalnych
mgr inż. Marcin Behnke

<u>Do wiadomości</u>:
- SDOO/ZDOO zainteresowane

<u>Załącznik</u>:
Wykaz odmian

Załącznik

Wykaz odmian kukurydzy w doświadczeniach porejestrowych 2012

Grupa wczesna

1. ES Cirrius
2. ES Kongress
3. ES Zizou
4. Laurinio
5. MAS 15P
6. Silvinio
7. SY Cooky
8. Nerissa*
9. NK Falkone*

Grupa średniowczesna

1. Ambrosini
2. ES Albatros
3. ES Palazzo
4. Konsulixx
5. NK Nekta
6. Prollix
7. Ricardinio
8. SY Multitop
9. LG 32.58*
10. Luigi CS*
11. Tiberio*

Grupa średniopóźna

1. Alduna
2. Amoroso
3. ES Chrono
4. Grosso
5. Lavena
6. Lindsey
7. MAS 29H
8. P9400
9. PR38N86
10. Ronaldinio
11. Castelli CS*
12. Crispi*
13. PR38A79*

* – odmiana z katalogu UE w doświadczeniach porejestrowych
